# TIMP-1 gene deficiency increases tumour cell sensitivity to chemotherapy-induced apoptosis

**DOI:** 10.1038/sj.bjc.6603378

**Published:** 2006-10-17

**Authors:** M L Davidsen, SØ Würtz, M U Rømer, N M Sørensen, S K Johansen, I J Christensen, J K Larsen, H Offenberg, N Brünner, U Lademann

**Affiliations:** 1 Department of Veterinary Pathobiology, The Royal Veterinary and Agricultural University, Ridebanevej 9, DK-1870 Frederiksberg C, Denmark; 2 Department of Surgical Gastroenterology, H:S Hvidovre Hospital, DK-2650 Hvidovre, Denmark

**Keywords:** TIMP-1, apoptosis, chemotherapy, fibrosarcoma cells

## Abstract

Tissue inhibitor of metalloproteinases-1 (TIMP-1) is one of four inhibitors of the matrix metalloproteinases, which are capable of degrading most components of the extracellular matrix. However, in recent years, TIMP-1 has been recognised as a multifunctional protein, playing a complex role in cancer. In this regard, several studies have demonstrated an antiapoptotic effect of TIMP-1 in a number of different cell types. Since chemotherapy works by inducing apoptosis in cancer cells, we raised the hypothesis that TIMP-1 promotes resistance against chemotherapeutic drugs. In order to investigate this hypothesis, we have established TIMP-1 gene-deficient and TIMP-1 wild-type fibrosarcoma cells from mouse lung tissue. We have characterised these cells with regard to TIMP-1 genotype, TIMP-1 expression, malignant transformation and sensitivity to chemotherapy-induced apoptosis. We show that TIMP-1 gene deficiency increases the response to chemotherapy considerably, confirming that TIMP-1 protects the cells from apoptosis. This is to our knowledge the first study investigating TIMP-1 and chemotherapy-induced apoptosis employing a powerful model system comprising TIMP-1 gene-deficient cells and their genetically identical wild-type controls. For future studies, this cell system can be used to uncover the mechanisms and signalling pathways involved in the TIMP-1-mediated inhibition of apoptosis as well as to investigate the possibility of using TIMP-1 inhibitors to optimise the effect of conventional chemotherapy.

The matrix metalloproteinases (MMPs) are a family of proteolytic enzymes, which are partly responsible for the turnover of the extracellular matrix in normal physiological conditions of tissue remodelling as well as in disease conditions such as invasion of cancer cells (Egeblad and Werb, 2002). Tissue inhibitor of metalloproteinases-1 (TIMP-1) is a naturally occurring inhibitor of the MMPs (Brew *et al*, 2000). It forms 1 : 1 stoichiometric complexes with the enzymes, thereby inhibiting the proteolytic activity of these molecules. Since the proteolytic activity of the MMPs is believed to facilitate invasion of cancer cells, one would expect TIMP-1 to inhibit tumour progression. However, a number of studies have demonstrated that the level of TIMP-1 is increased in several cancer forms, for example, colorectal and breast cancer and this increase has often been associated with a poor clinical outcome of the cancer patients (Ree *et al*, 1997; Holten-Andersen *et al*, 1999, 2000; McCarthy *et al*, 1999; Schrohl *et al*, 2004; Yukawa *et al*, 2004). This paradoxical finding has been suggested to be the consequence of distinct tumour-stimulating functions demonstrated for TIMP-1, for example, stimulation of proliferation (Hayakawa *et al*, 1992) and inhibition of apoptosis (Guedez *et al*, 1998; Li *et al*, 1999; Murphy *et al*, 2002; Lee *et al*, 2003; Liu *et al*, 2003, 2005; Boulday *et al*, 2004; Murphy *et al*, 2004).

The antiapoptotic effect of TIMP-1 induced by various apoptotic agents has been demonstrated in several different cell types such as human breast epithelial cells (Li *et al*, 1999; Liu *et al*, 2003, 2005), human breast carcinoma cells (Lee *et al*, 2003), human endothelial cells (Boulday *et al*, 2004), hepatic stellate cells (Murphy *et al*, 2002, 2004) and Burkitt's lymphoma cells (Guedez *et al*, 1998). Of these studies, only Li *et al* (1999) investigated the association between TIMP-1 level and cell survival following treatment with chemotherapy (adriamycin). However, a specific event of apoptosis was not confirmed in this survival experiment. In support of an antiapoptotic function of TIMP-1, our laboratory has recently shown that in patients with metastatic breast cancer, the response to chemotherapy was 0% in patients with primary tumours containing high levels of TIMP-1, while being 45% in patients with tumours containing low levels of TIMP-1 supporting a protective role of TIMP-1 to chemotherapy-induced apoptosis (Würtz *et al*, 2005). Similar results have also been demonstrated for metastatic colorectal cancer (Sørensen *et al*, 2006).

Taken together, this raises the hypothesis that TIMP-1 protects cells against chemotherapeutic treatment by inhibiting apoptosis. In order to investigate this hypothesis, we have established TIMP-1 gene deficient and TIMP-1 wild-type fibrosarcoma cell lines from lung tissue originating from littermate mice. We have confirmed these two different TIMP-1 variants by PCR, RT–PCR, Western blotting and ELISA. We have demonstrated that the cells display immortalised and clonogenic growth, showing that the cells had spontaneously transformed to a malignant phenotype. In addition, we have analysed the sensitivity of the cells to chemotherapy-induced apoptosis and shown that TIMP-1 gene-deficient cells are considerably more sensitive to induced apoptosis compared to the corresponding TIMP-1 wild-type cells. This confirms that TIMP-1 plays a role in inhibition of chemotherapy-induced apoptosis in the established cell lines. This cell system represents a powerful tool for further studies of the antiapoptotic role of TIMP-1 in cancer, especially considering the quality of having a model system consisting of gene deficient cells and their identically genetic wild-type control. Furthermore, this cell system can be used to uncover the mechanisms and signalling pathways involved in the TIMP-1-mediated inhibition of apoptosis. Evidence from a number of recent studies suggests that the focal adhesion kinase (FAK)/phosphatidylinositol-3 kinase (PI-3 kinase)/Akt/Bad/Bcl-X_L_/Bcl-2 signalling pathway is involved in the TIMP-1-mediated inhibition of apoptosis (Guedez *et al*, 1998; Li *et al*, 1999; Lee *et al*, 2003; Liu *et al*, 2003, 2005; Boulday *et al*, 2004); however, further studies are still needed to identify the mechanisms involved.

Importantly, as suggested by the present study, TIMP-1 may be a new therapeutic target in the optimisation of treatment with conventional anticancer drugs and future studies using this cell system offers the opportunity to investigate this hypothesis.

## MATERIALS AND METHODS

### Compounds

Etoposide (Bristol-Myers Squibb, Denmark), cytosar (Pfizer, Denmark) and vincristine (Faulding, Denmark) were kindly provided by Peter Buhl Jensen (Rigshospitalet, Denmark).

### Establishment of cell cultures from transgenic mice

TIMP-1 gene-deficient and wild-type mice were kindly provided by Paul D Soloway, Cornell University (Ithaca, NY, USA). In brief, TIMP-1 gene-deficient mice were generated by homologous recombination of a neomycin-containing TIMP-1 insertion vector in mouse embryonic stem cells. Stem cells were then injected in blastocysts and transgenic mice were generated by backcrossing into the BALB/cJ mouse strain. Genotypes of the mice were determined by PCR on genomic tail DNA (Soloway *et al*, 1996). Establishment of cell cultures was performed as described previously (Rømer *et al*, 2005). In brief, lungs of TIMP-1 gene-deficient and wild-type littermate mice (29 weeks old males) were excised and placed in Petri dishes containing 10 ml of medium (M199, Gibco, Invitrogen A/S, Denmark) supplemented with 30% foetal calf serum (FCS), penicillin (100 units/ml), streptomycin (100 *μ*g/ml) and 1% NaHCO_3_ (Gibco, Invitrogen A/S, Denmark). The lungs were cut into small pieces and 3–6 pieces/well were placed in a six-well plate (Nunc, Denmark) together with a drop of medium and incubated at 5% CO_2_ and 37°C for 20 min to allow the cells to adhere. After 20 min, 1 ml of medium was added to completely cover the tissue. After additional 30 min, another 1 ml of medium was added. The medium was renewed every third day. The wells were inspected regularly during the next 3 weeks and thereafter the medium added was without penicillin and streptomycin. After 4–5 weeks, the wells with outgrowth of fibroblasts were harvested and cells from the same mouse were pooled. The medium was changed to M199 medium with 10% FCS and 0.15% NaHCO_3_ (CM). A total of four fibrosarcoma cell lines were successfully established. These cell lines were named Twt-II and Twt-III (TIMP-1 wild type) and Tko-II and Tko-III (TIMP-1 gene deficient), respectively. Within each pair (II and III), the Twt and Tko cells were derived from littermates in order to assure identical genetic background. The Twt-II and Tko-II were established 4 months apart from the Twt-III and Tko-III cell lines. The cells were tested for infectious agents in the Mouse RapidMAP18 panel (Taconic Europe) all with negative results.

### Genotyping

The cells (passages 60–69) were trypsinised, harvested and DNA was isolated as described previously (Laird *et al*, 1991). One microlitre of each DNA sample was transferred to 25 *μ*l PCR-working solution (1 × Hot Star Tag Mastermix (Qiagen, Ballerup, Denmark), MgCl_2_ (25 mM) and 2 *μ*l of each primer: forward primer recognising Twt: 5′-CAAGGGGTGCTTAGTTGCTCTGG-3′ (exon 3); reverse primer recognising Twt: 5′-CACTGCGGTTCTGGGACTTGTGG-3′ (exon 4); forward primer recognising Tko: 5′-GCGAACCCACCTCTCCGATAAGC-3′ (neo-cassette) and reverse primer recognising Tko: 5′-CATTCGCCGCCAAGCTCTTCAGC-3′ (neo-cassette). The expected size of the Twt-specific PCR product is approximately 370 base pairs (bp), whereas the Tko-specific PCR product is approximately 670 bp. The reaction conditions optimised for the two genotype-specific bands were 95°C for 15 min, followed by 32 cycles of 94°C for 1 min, 63°C for 1 min, 72°C for 1 min and finally 72°C for 10 min. In each assay, three controls were included: TIMP-1 +/0, TIMP-1 −/0 (TIMP-1 is located on the X-chromosome and only male mice were employed in the experiments; Huebner *et al*, 1986) and as negative control H_2_O. Tissue inhibitor of metalloproteinases-1 +/0 and TIMP-1 −/0 control samples were obtained by enzymatic digestion of tail tissue from TIMP-1 wild-type and TIMP-1 gene-deficient mice, respectively. The PCR products were run on a 2% agarose gel (Fermentas, Helsingborg, Sweden), stained with ethidium bromide and visualised by UV light.

### RT–PCR

RNA was extracted from the cells (passages 72–78) using an SV Total RNA isolation kit (Promega, Manheim, Germany). The cells were grown in 10 cm culture dishes to a confluence of 90% and lysed in 500 *μ*l lysis buffer. RNA from 175 *μ*l of the lysed cells was extracted following the manufacturer's instructions. DNase treatment was included in the protocol to avoid genomic DNA contamination of the RNA. Concentration of the RNA was estimated by measuring OD_260_. Two micrograms of RNA was reverse transcribed to cDNA in 20 *μ*l RT buffer (Fermentas) containing 1 mM dNTPs, 20 U RiboLock RNase inhibitor, 0.5 *μ*g oligo(dT)primer, 0.2 *μ*g random hexamer primer and 40 U M-MuLV Reverse Transcriptase. Samples were incubated at 25°C for 10 min, followed by 42°C for 1 h. The reaction was terminated by incubation at 95°C for 5 min followed by cooling on ice. To ensure that RNA extractions and RT reactions were successful, PCR using intron-spanning *β*-actin primers was performed on all cDNA samples (forward primer: 5′-CGTGGGCCGCCCTAGGCACCA-3′ and reverse primer: 5′-TTGGCCTTAGGGTTCAGGGGGG-3′). The expected size of the *β*- actin PCR product is 242 bp. To test for expression of TIMP-1 mRNA from Twt and Tko cells, PCR using intron-spanning TIMP-1-specific primers was carried out on cell cDNA (forward primer: 5′-GTGGGAAATGCCGCAGATATC-3′ and reverse primer: 5′-GACCTGATCCGTCCACAAAC-3′). The expected size of this PCR product is 299 bp. PCR was performed in 25 *μ*l reactions consisting of 1 × Hot Star Taq Mastermix, 1 *μ*M of each gene-specific primer and 1 *μ*l cDNA. Reaction conditions were 95°C for 15 min followed by 40 cycles of 94°C for 1 min, 60°C for 30 s and 72°C for 1 min. PCR products were run on a 1.5% agarose gel (Fermentas), stained with ethidium bromide and visualised by UV light.

### Western blotting

Cells were seeded in 10 cm Petri dishes in CM. When about 90% confluent, the cells were harvested by scraping and resuspended in 100 *μ*l lysis buffer (0.5% Triton X-100, 25 mM Hepes1, 5 mM MgCl_2_, 1 mM EGTA, milliQ H_2_O) containing four protease inhibitors (Aprotinin, Leupeptin, Pepstatin A and Pefa Block in a final concentration of 1 *μ*g/ml). The protein concentration in each sample was determined by protein BCA Protein Assay kit (Pierce, Rockford, IL, USA). Proteins were separated by sodium dodecyl sulphate (SDS)–gel electrophoresis using a 12% polyacrylamide gel and blotted on nitrocellulose paper. The blot was blocked in washing buffer (phosphate-buffered saline (PBS)+0.1% Tween 20) containing 5% dry milk and incubated with the rat-IgG antibody to murine TIMP-1 (R&D Systems, UK, 3 *μ*g/ml in washing buffer containing 1% dry milk) or the goat-IgG antibody to murine p53 (R&D Systems, diluted 1 : 2000 in washing buffer containing 1% dry milk) or rat-IgG antibody to murine p19^Arf^ (Upstate, Charlottesville, USA, diluted 1 : 100 in washing buffer containing 1% dry milk) for TIMP-1, p53 and p19^Arf^ detection, respectively. Subsequently, the blot was washed 3 × 10 min in washing buffer followed by incubation with the appropriate horseradish peroxidase-conjugated secondary antibody (rabbit-IgG to rat-IgG diluted 1 : 1000 or rabbit-IgG to goat-IgG diluted 1 : 10 000 or rabbit-IgG to rat-IgG diluted 1 : 2000 (DAKO, Denmark), respectively). Following 3 × 10 min washes in washing buffer, the blot was developed by the ECL detection system (Amersham Bioscience, UK) according to the manufacturer's instructions. In order to obtain a loading control, the blot was stripped and re-probed with a primary monoclonal antibody recognising glyceraldehyde-3-phosphate dehydrogenase (GAPDH) (Biogenesis, UK, diluted 1 : 80 000 in washing buffer containing 1% dry milk). Subsequently, the blot was washed 3 × 10 min in washing buffer and incubated with a polyclonal horseradish peroxidase-conjugated goat-IgG to mouse-IgG (DAKO, Denmark, diluted 1 : 100 000 in washing buffer containing 1% dry milk) for 1 h. Finally, the blot was washed 3 × 10 min in washing buffer and developed as described above. Details regarding cells are indicated in the figure legends.

### Murine TIMP-1 ELISA

Cells (2500 cells/well, passages 55–56) were seeded in 96-well microtitre plates in triplicate in 100 *μ*l CM. After 24 h, the CM was replaced with 150 *μ*l new CM. After another 24 h, the CM was collected, centrifuged for 5 min at 300 **g** and the supernatant transferred to Eppendorf tubes and stored at −20°C. The amount of TIMP-1 secreted in the CM was measured using Quantikine Mouse TIMP-1 Immunoassay (R&D Systems, UK) according to the manufacturer's instructions.

### Clonogenic assay

Clonogenic assay was performed as described previously (Jensen *et al*, 1993). In brief, cells (passages 27–35) were suspended in CM supplemented with penicillin (50 U/ml) and streptomycin (50 *μ*g/ml). A volume of 350 *μ*l cell suspension was mixed with a mixture of agar and medium. One microlitre of the cell-containing agar was then plated in triplicate in Petri dishes over a layer of sheep red blood cells. When the agar had solidified, 1 ml of media were added on top. Cells were grown in a CO_2_ incubator (7.5% CO_2_) at 37°C and 100% humidity. Following 3 weeks of incubation, colonies (>64 cells) were counted using the software Sorcerer (Perceptive Instruments, Suffolk, UK).

### Flow cytometrical analysis

DNA ploidy of early (passages 29–38), middle (passages 45–48) and late passages (passages 62–71) was determined for each cell line by flow cytometrical analysis as described previously (Vindeløv and Christensen, 1990; Rømer *et al*, 2005). All measurements were related to the DNA index of normal diploid mouse cells.

### Cell death assays

#### Lactate dehydrogenase (LDH) release assay

To investigate the response to chemotherapy in the cell lines, cytotoxicity was determined following treatment with chemotherapeutic drugs. During cell culture conditions, cells that have been given an apoptotic stimulus will initially die by apoptosis and later turn into secondary necrosis due to the lack of phagocytosis. Cytotoxicity or cell lysis can be measured by the release of LDH in the culture supernatant. To measure the LDH release, the Cytotoxicity Detection Kit (Roche, Hvidovre, Denmark) was employed. The cells (passages 39–87) were seeded in 96-well microtitre plates (3000 cells/well). After 24 h, the cells were treated with the chemotherapeutic drug. After 48 h (etoposide) or 35 h (cytosar and vincristine) incubation, 50 *μ*l (out of 200 *μ*l) of culture supernatant was transferred to a new 96-well microtitre plate and mixed with 50 *μ*l of substrate mix. The remaining culture supernatant was discarded and the residual intact adherent cells were lysed by the addition of 200 *μ*l lysis buffer (1% Triton X-100 in CM). Following lysis for 30 min at 5% CO_2_ and 37°C, 50 *μ*l of the lysate was transferred to a new 96-well microtitre plate and mixed with 50 *μ*l of substrate mix. Both the cell culture supernatants and the lysates were incubated with substrate mix for 10 min at room temperature protected from light. The absorbance was measured in a spectrophotometer at *λ*_1_=570 nm and reference *λ*_2_=630 nm. The amount of released LDH in per cent was related to the total amount as follows: 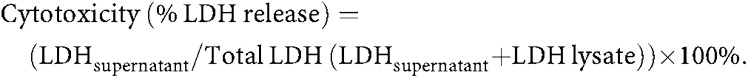


#### Apoptosis assay

To confirm that the cell death observed in the LDH assay was apoptotic, the presence of DNA–histone complexes in the cytoplasm following the apoptotic stimuli was measured. For this purpose, the Cell Death Detection ELISA Kit (Roche) was employed. The cells (passages 39–87) were seeded in a 96-well microtitre plates (3000 cells/well). After 24 h, the cells were treated with the chemotherapeutic drug for 48 h (etoposide) or 35 h (cytosar and vincristine) and the level of apoptosis was measured according to the manufacturer's instructions. The degree of apoptosis (DNA fragmentation) was calculated as follows: absorbance of sample (dying and dead cells)/absorbance of corresponding control (viable cells).

### Cell proliferation assay

Cell proliferation was estimated using the CyQuant® Cell Proliferation Assay Kit (Molecular Probes Inc., Eugene, OR, USA/Invitrogen, Denmark). Cells (500 cells/well, passages 49–51 and passages 81–86) were seeded in 96-well microtitre plates and harvested daily over a 5- or 6-day period. Proliferation rates were analysed by spectrophotometrical measurements of the DNA and RNA content at 480 nm excitation and 520 nm emission according to manufacturer's instructions.

## RESULTS

### TIMP-1 genotype and TIMP-1 expression

**Figure 1:**
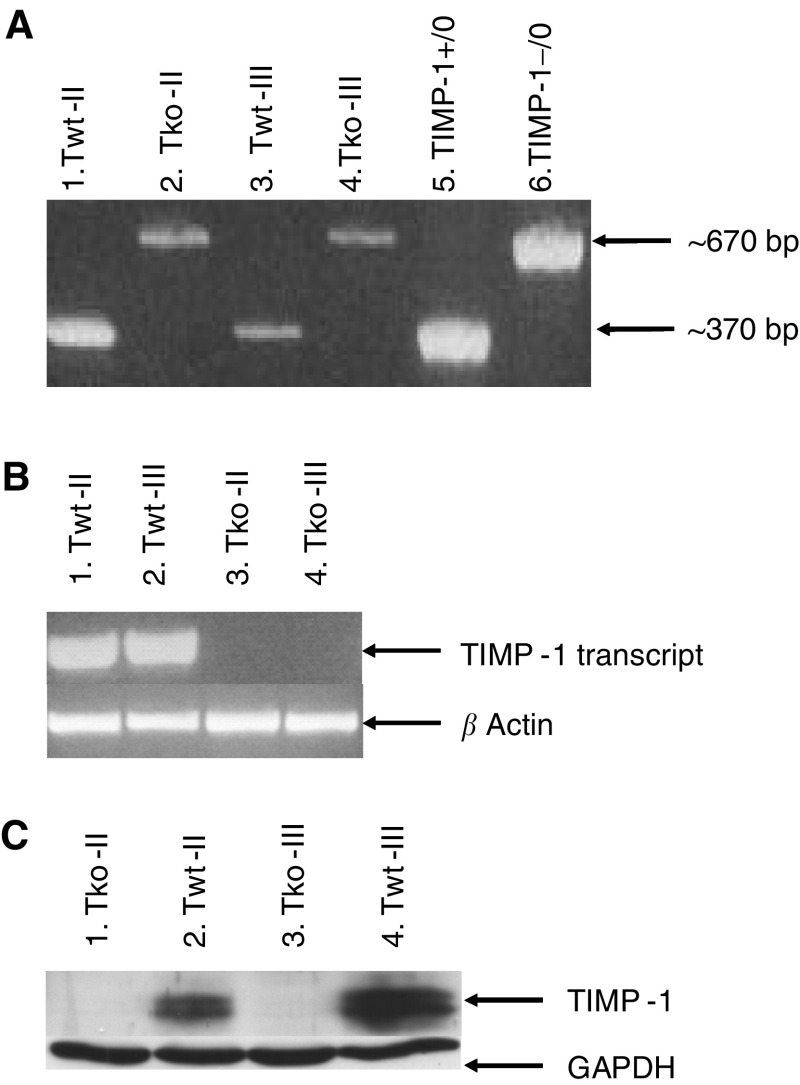
Confirming the absence or presence of TIMP-1 in the gene-deficient and wild-type fibrosarcoma cell lines respectively. (**A**) Genotyping. Lane 1: Twt-II in passage 60; lane 2: Tko-II in passage 60; lane 3: Twt-III in passage 65; lane 4: Tko-III in passage 65; lane 5: +/0 TIMP-1 control; and lane 6: −/0 Timp-1 control. The PCR was performed on lysate from the cells, each sample and each control with both sets of primers. (**B**) RT–PCR. Lane 1: Twt-II in passage 73; lane 2: Twt-III in passage 78; lane 3: Tko-II in passage 72; and lane 4: Tko-III in passage 78. The RT–PCR has been run on lysate from the cells, each sample with intron-spanning *β*-actin primers and with intron-spanning TIMP-1-specific primers. (**C**) Western blotting. Lane 1: Tko-II in passage 74; lane 2: Twt-II in passage 68; lane 3: Tko-III in passage 74; and lane 4: Twt-III in passage 70. In each lane, equal amounts of protein (60 *μ*g protein) are loaded. The presence of two bands may reflect two different glycosylation products of TIMP-1.

In order to confirm the absence or presence of TIMP-1 in the TIMP-1 gene-deficient and the TIMP-1 wild-type cells, respectively, genotyping, RT–PCR, Western blotting and ELISA were performed (Figure 1). Genotypes of the TIMP-1 wild-type and TIMP-1 gene-deficient cells were determined by PCR (Figure 1A). As expected, the wild-type control (TIMP-1 +/0) gave rise to a PCR product of approximately 370 bp representing the wild-type TIMP-1 gene (Figure 1A, lane 5). Bands of the same sizes appeared when PCR was performed on lysates from the Twt-II and Twt-III cell lines, thereby confirming that both of these cell lines exhibit the wild-type TIMP-1 gene (Figure 1A, lanes 1 and 3). In contrast, the PCR of the TIMP-1 gene-deficient control (TIMP-1 −/0) resulted in a PCR product of approximately 670 bp, reflecting disruption of exon 4 in the TIMP-1 gene by insertion of a neo-cassette (Figure 1A, lane 6). The band of 670 bp was also present in the Tko-II and Tko-III cell lines confirming the gene-deficient status of these cells. To ensure that the TIMP-1 gene is only transcribed in the TIMP-1 wild-type cells and not in the TIMP-1 gene-deficient cells, RT–PCR assay of cell lysates from the Twt-II, Twt-III as well as the Tko-II and Tko-III cell lines was performed (Figure 1B). As Figure 1B shows, only the wild-type cells gave rise to a TIMP-1 mRNA transcript (compare lanes 1 and 2 with lanes 3 and 4). Using *β*-actin primers resulted in a PCR product of the expected size in all samples, confirming the presence of cDNA (Figure 1B). To investigate whether the expression of TIMP-1 observed in the wild-type, but not in the gene-deficient cells also applied to TIMP-1 protein, Western blotting was performed (Figure 1C). As seen in the figure, only the Twt cells expressed the TIMP-1 protein (lanes 2 and 4), whereas the Tko cells did not (lanes 1 and 3). In addition, it can be seen that the Twt-III has an overall increased level of TIMP-1 expression compared to the Twt-II cells. Likewise, ELISA measurements showed a considerably higher amount of TIMP-1 protein in CM from Twt-III cells (∼8.6 ng/ml)) compared to Twt-II cells (∼2.2 ng/ml), and no TIMP-1 was detected in either of the TIMP-1 gene-deficient cell lines.

### Transformation of the cells

The cells were analysed to establish whether they had spontaneously transformed to a malignant phenotype. All four cell lines displayed immortalised growth since they survived in culture at least to passage 120. More importantly, the clonogenic assay demonstrated that all cell lines were able form colonies in soft agar (data not shown).

**Figure 2:**
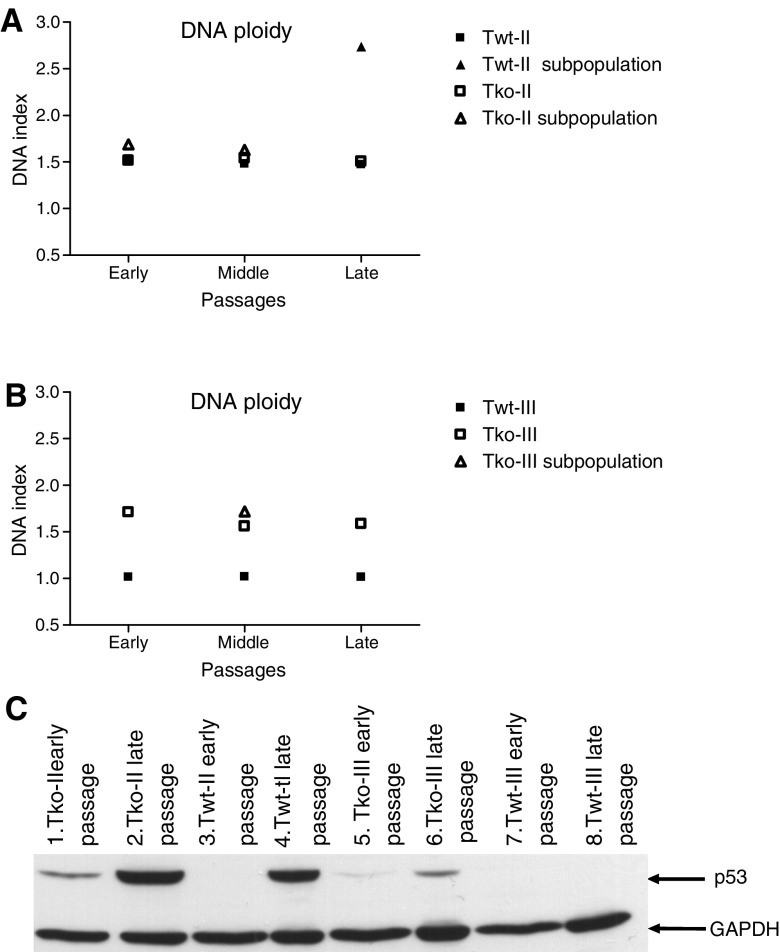
Transformation of the cell lines. (**A**, **B**) DNA ploidy analysis of Twt-II and Tko-II and Twt-III and Tko-III, respectively, in early, middle and late passages. A DNA index of 1 represents normal diploid mouse DNA content. (**C**) Western blot of p53 expression in the fibrosarcoma cells. Lane 1: Tko-II in passage 11; lane 2: Tko-II in passage 69; lane 3: Twt-II in passage 14; lane 4: Twt-II in passage 68; lane 5: Tko-III in passage 12; lane 6: Tko-III in passage 74; lane 7: Twt-III in passage 10; and lane 8: Twt-III in passage 70. In each lane equal amounts of protein (40 *μ*g) are loaded.

Loss of euploidy is a characteristic of the transformation process and therefore we investigated DNA ploidy of the cells (Figure 2A and B). This analysis showed that both Twt-II and Tko-II cells were aneuploid in all the investigated passages according to their DNA indices of approximately 1.5. Both cell lines had subpopulations in different passages (Figure 2A). Likewise, the Tko-III cells were aneuploid, having a DNA index of approximately 1.6 with a single subpopulation. In contrast, the Twt-III cells were diploid with a DNA index of 1 in all passages tested (Figure 2B). Disruption of the p53 signalling pathway is also a frequent event in the transformation process. This typically occurs by an inactivation mutation in the p53 gene or inactivation of the tumour suppressor p19^Arf^, a positive regulator of the p53 gene. Inactivation of p53 directly leads to an increase in the expression level of the protein (Soussi and Béroud, 2001). Western blotting showed a pronounced increase in the expression level of p53 in late passages (>passage 68) compared to early passages (<passage 14) of Twt-II, Tko-II and Tko-III cells (Figure 2C). In contrast, the immunoblot analysis of the Twt-III cells revealed no detectable levels of p53 in either early or late passages. Thus, this further supports that the Twt-II, Tko-II and Tko-III cells have been malignantly transformed. The fact that there was no indication of inactivation of the p53 pathway in the Twt-III cells and that the cells were diploid implies that a different transformation process had taken place in this cell line. To analyse this further, we investigated the level of the tumour suppressor p19^Arf^. As determined by Western blotting, there was no expression of p19^Arf^ in late passages of Twt-III cells, indicating that an indirect disruption of the p53 signalling pathway had taken place, thereby supporting that these cells had been malignantly transformed, although through a different process compared to the other cell lines (data not shown).

### TIMP-1 gene deficiency confers increased sensitivity to chemotherapeutic drugs

**Figure 3:**
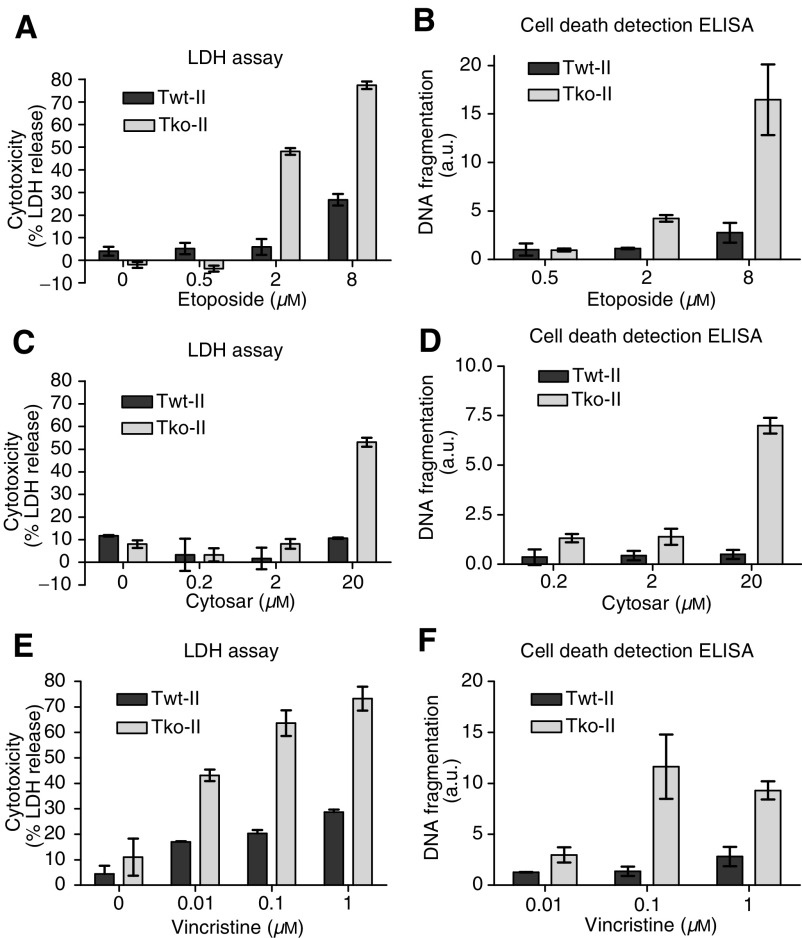
Sensitivity to chemotherapeutic drugs. Twt-II and Tko-II cells were treated with etoposide (**A**, **B**) for 48 h or cytosar (**C**, **D**) or vincristine (**E**, **F**) for 35 h. Induced cell death (**A**, **C**, **E**) and induced apoptosis (**B**, **D**, **F**) are depicted for all three drugs. The experiments were repeated more than three times through passages 39–87 with same results. This figure is representative of all experiments performed. Values represent means of triplet determination±s.d.

TIMP-1 has been demonstrated to inhibit apoptosis induced by various stimuli in several different types of cells (Guedez *et al*, 1998; Li *et al*, 1999; Murphy *et al*, 2002; Lee *et al*, 2003; Liu *et al*, 2003, 2005; Boulday *et al*, 2004; Murphy *et al*, 2004). In order to investigate whether TIMP-1 inhibits chemotherapy-induced apoptosis, we tested the sensitivity of the TIMP-1 wild-type and the TIMP-1 gene-deficient cells to treatment with three different chemotherapeutic drugs and the cytotoxicity was measured by LDH release assay (Figure 3). In Figure 3A, the results from treatment of the Twt-II and Tko-II cell lines with the drug etoposide are shown. As seen in the figure, both cell lines displayed a dose-dependent response to the treatment; however, the TIMP-1 gene-deficient cells were considerably more sensitive to the treatment compared to the wild-type cells. To confirm that the cell death induced by etoposide was apoptotic, we determined the level of DNA–histone complexes in the cytoplasm following drug treatment. As is evident from Figure 3B, the cell death induced by etoposide was indeed apoptotic. The experiments with etoposide were also conducted with Twt-III and Tko-III with the same results (data not shown) showing that the TIMP-1-mediated protection was not dependent on the transformation process. We also tested the effect of the two chemotherapeutic drugs, cytosar and vincristine, on the Twt-II and Tko-II cell lines. The results from the LDH release assay as well as the apoptosis assay are shown in Figure 3C–F. As was the case concerning etoposide, the Tko-II cells were considerably more sensitive to the drugs tested compared to the Twt-II cells as determined by LDH release assay (Figure 3C and E). In addition, the apoptosis assay confirmed that the enhanced cell death observed in the Tko-II cells was a result of increased apoptosis (Figure 3D and F). Together, these results demonstrate that TIMP-1 deficiency increases the cellular response to chemotherapy-induced apoptosis.

### Similar growth rates of the TIMP-1 gene-deficient and wild-type cells

**Figure 4:**
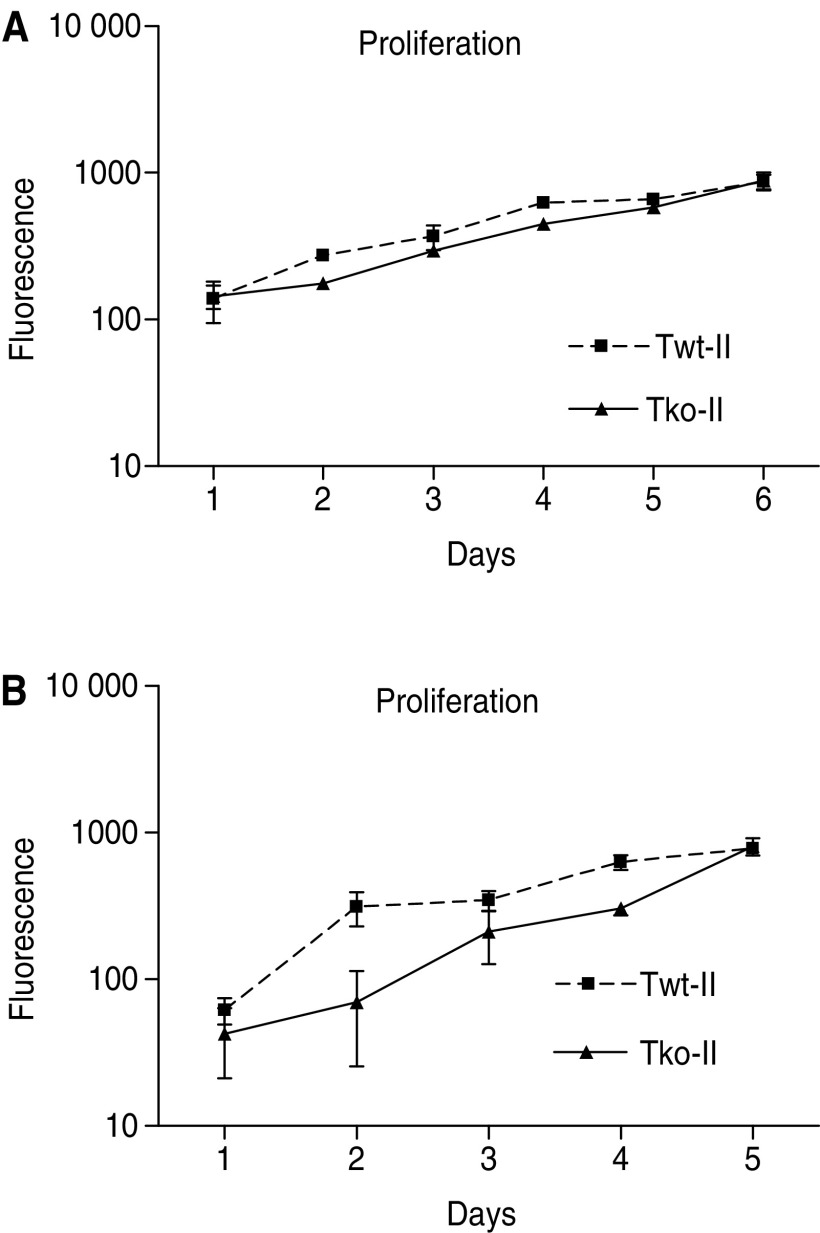
Proliferation. Growth rates of Twt-II and Tko-II were investigated by a proliferation assay. Fluorescence reflects the DNA content. (**A**) Twt-II and Tko-II in passages 49–51. (**B**) Twt-II and Tko-II in passages 81–82.

In general, chemotherapeutic drugs induce apoptosis in cancer cells by disturbance of cell division through damaging of the DNA. Therefore, rapidly dividing cells are affected more by chemotherapy than normally dividing cells. To ensure that the differential response to chemotherapy observed in the cell lines is not the result of different growth rates, we investigated the doubling times of the cells using a cell proliferation assay (Figure 4). In passages 49–51, the doubling time for Twt-II was 1.96 days and for Tko-II 1.86 days (Figure 4A), and in passages 81–86, the doubling times was 1.20 and 0.94 days (Figure 4B), respectively, confirming similar proliferation rates of the TIMP-1 gene-deficient and wild-type cells. Thus, the difference in sensitivity to induced apoptosis cannot be ascribed to a difference in growth rates. Similar growth rates were also found for Twt-III (passages 49–51: 1.28 days; passage 81–86: 1.21 days) and Tko-III (passages 49–51: 1.94; passage 81–86: 1.41 days).

## DISCUSSION

It is well established that TIMP-1 is able to inhibit apoptosis induced by various apoptotic stimuli in a number of different cell types. A single study has suggested that TIMP-1 enhances survival during chemotherapeutic treatment (Li *et al*, 1999). In addition, recent clinical studies demonstrated a significant association between high tumour tissue levels of TIMP-1 and no objective responses to the most frequently used chemotherapeutic drugs for patients with metastatic breast cancer and colorectal cancer, indicating that TIMP-1 also protects against chemotherapy-induced apoptosis *in vivo* (Würtz *et al*, 2005; Sørensen *et al*, 2006).

In an attempt to obtain a system, which can be used for further investigations of the protective function of TIMP-1 against chemotherapy-induced apoptosis, we have established TIMP-1 wild-type and TIMP-1 gene-deficient fibrosarcoma cell lines from lung tissue originating from littermate mice. We have characterised these cells and confirmed the two different TIMP-1 variants by PCR, RT–PCR, Western blotting and ELISA. Moreover, we have assured that the cells have been malignantly transformed by demonstration of immortalised and clonogenic growth as well as aneuploidy and/or disruption of the p53 signalling pathway. It should be emphasised that this new well-characterised cell system has the strength of exhibiting two pairs of cell lines sharing, except from TIMP-1, identical genetic backgrounds, making it possible to investigate the isolated function of TIMP-1.

To investigate whether TIMP-1 could play a role in inhibition of chemotherapy-induced apoptosis in our cell system, we tested the response of the cells to apoptosis induced by three different chemotherapeutic drugs. We showed that the TIMP-1 gene-deficient fibrosarcoma cells were considerably more sensitive to the chemotherapeutic drugs tested, compared to the corresponding TIMP-1 wild-type cells, confirming that TIMP-1 protects against chemotherapy in our model system. Interestingly, the higher level of TIMP-1 expression observed in the Twt-III cell line compared to the Twt-II cell line is not associated with increasing protection against apoptosis, suggesting that both wild-type cell lines have reached a saturation level of TIMP-1 concerning the protection against apoptosis (data not shown). Furthermore, the protection seems independent of the mechanism by which apoptosis was induced, since TIMP-1 protected the cells from apoptosis induced by three different drugs (etoposide, cytosar and vincristine) working through different DNA-damaging mechanisms (inhibitor of topoisomerase II, inhibitor of DNA repair and DNA polymerase and an antimicrotubule agent, respectively) (Holland *et al*, 2000). As the differential response to chemotherapy could be the result of different growth rates in the cell lines, we analysed the proliferation rates of the cells. We found that the TIMP-1 wild-type and the TIMP- 1 gene-deficient cells had similar growth rates, confirming that the difference in response to apoptosis-inducing drugs was not the result of a difference in growth, but was coursed by inhibition of apoptosis in the wild-type cells. Interestingly, a previous study completed in our laboratory has revealed a similar association between plasminogen activator inhibitor-1 (PAI-1) gene deficiency and increased sensitivity to chemotherapy-induced apoptosis (Lademann *et al*, 2005; Rømer *et al*, 2005), suggesting an overlapping role of TIMP-1 and PAI-1. Therefore, we have established fibrosarcoma cells lines simultaneously deficient of the TIMP-1 and the PAI-1 genes, which can be used to investigate possible common characteristics of these two inhibitors (currently under investigation).

In summary, the cell systems established in this study have been thoroughly characterised with regard to TIMP-1 genotype, TIMP-1 expression, malignant transformation and response to chemotherapy. Our studies demonstrated that TIMP-1 deficiency confers a pronounced increase in sensitivity to apoptosis induced by chemotherapy, confirming that TIMP-1 plays a role in inhibition of apoptosis. Addition of exogenous TIMP-1 or reintroduction of the TIMP-1 gene in the gene-deficient cell lines, as well as inhibition of TIMP-1 expression in the wild-type cell lines, would further validate the specific antiapoptotic function of TIMP-1.

The established cell lines represent very important tools for future investigations of the antiapoptotic function of TIMP-1, especially considering the quality of having a model system, which includes the gene-deficient cells and their identical genetic wild-type controls, together with the possibility of conducting *in vivo* experiments. These include studying the effect of chemotherapeutic drugs on tumours induced by inoculation of our cells in the TIMP-1 gene-deficient and wild-type BALB/cJ mouse strain.

Finally, this model system can also be employed in the search for a putative TIMP-1 binding protein and the investigations of signalling pathways in which TIMP-1 may be involved. Obvious candidate molecules to be investigated are the molecules included in the survival pathway such as FAK, PI-3 kinase, Akt and Bcl-2 family members, previously shown to be regulated by TIMP-1. Importantly, the experiments performed suggest that TIMP-1 inhibitors could represent a novel treatment as a chemosensitising approach given before conventional therapy and the model system presented here offers the opportunity to investigate this hypothesis.
